# Repurposing dimethyl fumarate for cancer therapy: current evidence and future directions

**DOI:** 10.3389/fphar.2025.1721876

**Published:** 2025-12-01

**Authors:** Mingjuan Zhang, Yaping Jing, Qingbin Cui

**Affiliations:** 1 Guangzhou Vocational University of Science and Technology, Guangzhou, Guangdong, China; 2 School of Public Health, Guangzhou Medical University, Guangzhou, Guangdong, China; 3 Department of Pharmaceutical Sciences, College of Pharmacy and Health Sciences, St. John’s University, Queens, NY, United States

**Keywords:** Dimethyl fumarate, drug repurposing, anticancer, sensitizer, combination

## Abstract

Dimethyl fumarate (DMF) is an approved medication by the FDA for the treatment of multiple sclerosis, primarily targeting and regulating the NF-κB pathway. Recently, its anticancer effects have drawn considerable attention as it not only effectively kills a panel of different cancer cells *in vitro* and *in vivo*, but also synergizes with other conventional or targeted chemotherapeutics in certain resistant or refractory cancer cells. Mechanism studies showed that in addition to inhibiting NF-κB and stimulating Nrf2, DMF functioned as a chemotherapy also by suppressing inflammation, inhibiting epigenetic modifications, as well as modulating epithelial-mesenchymal transition (EMT). On the molecular level, DMF can form a covalent bond with the thiol group of a protein. In this paper, we provide a brief review of the anticancer studies of DMF, either as a single agent or in combination regimens. While DMF is a relatively weak cytotoxic agent, it is effective in sensitizing cells to other chemotherapeutic agents. Since DMF is already an approved drug, its fast-track approval for cancers may bring new hope to those chemo-resistant patients who suffer from very limited treatment options.

## 1 Introduction

Drug resistance remains a significant threat to cancer patients, since almost all chemotherapies, including the most recent immunotherapies, will eventually become less or non-responsive in pretreated patients (Kingwell, 2019). In addition, drug resistance is always involved multiple factors, rendering it a real conundrum (Assaraf et al., 2019; Cui et al., 2022; Narayanan et al., 2020). New drugs that are potent in suppressing pretreated and resistant cancers are urgently needed. While multiple novel technical developments, such as artificial intelligence (AI), large-scale proteomics, RNA-seq, etc., have significantly shortened the timeline and enhanced the chances of success in launching a new drug, it remains a time- and money-consuming business (Van de Sande et al., 2023; Mullowney et al., 2023). Other conventional strategies such as drug repurposing, are still a feasible and practical way in both academic and industrial pharmaceutical companies to develop new drugs (Gao et al., 2023; Cui et al., 2022).

**FIGURE 1:**
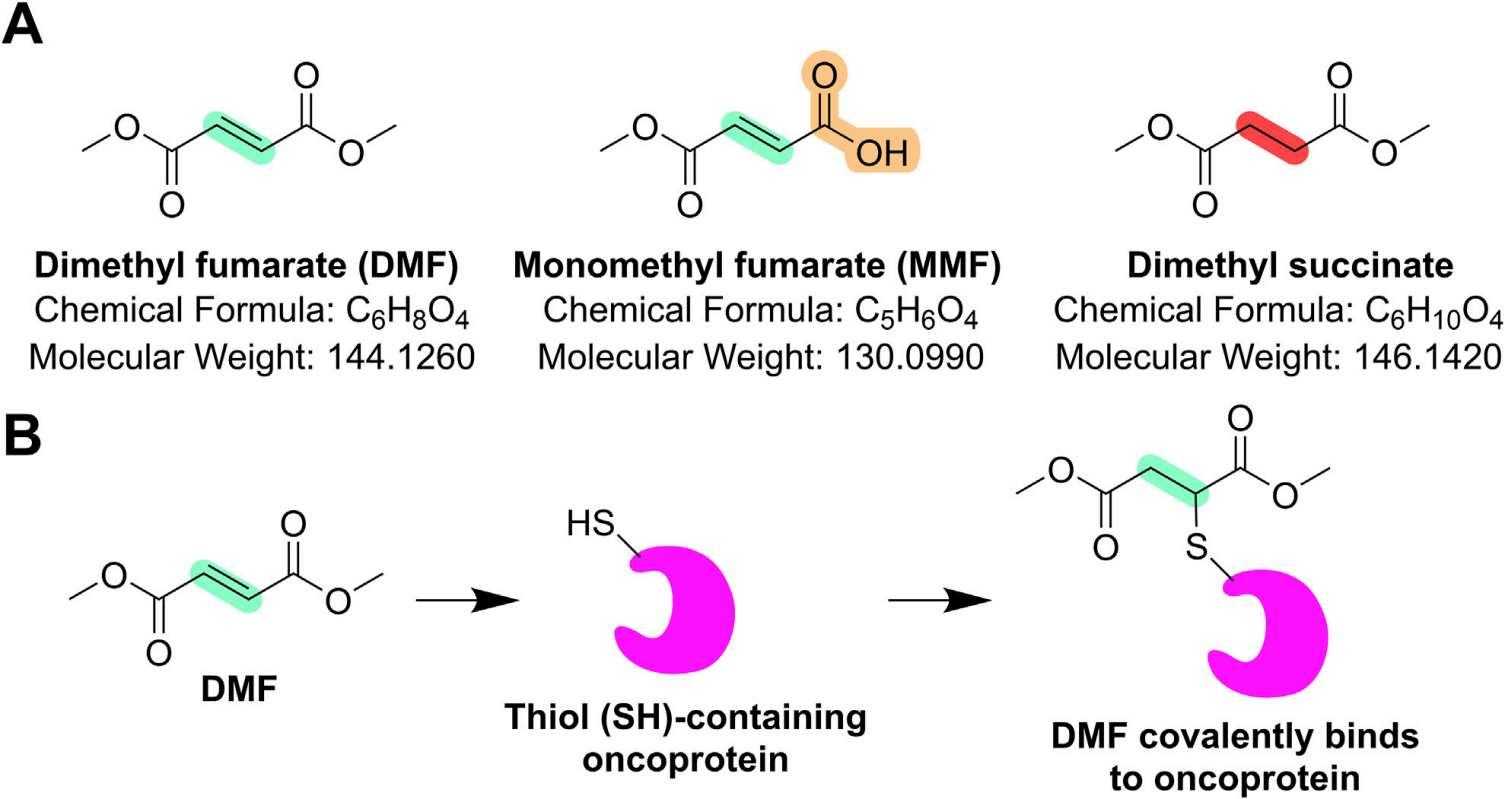
**(A)** DMF, its active metabolite MMF, and its analog dimethyl succinate, which lacks a double bond. The double bond in DMF, the free carboxyl group in MMF, and the single bond in dimethyl succinate were highlighted. **(B)** The double bond of DMF is chemically reactive and can undergo the Michael addition with the thiol group (–SH) of an oncoprotein, thereby blocking its functions in promoting cancers. However, dimethyl succinate, due to the lack of a double bond, is chemically and pharmacologically inactive and is usually used as a negative control for DMF.

Dimethyl fumarate (DMF), a methyl ester of fumaric acid as shown in Figure 1A, has a long-standing medical history that began in Germany in the 1950s, where it was first explored as a treatment for psoriasis (Deeks, 2016). The compound gained significant clinical relevance when a mixture of fumaric acid esters, including DMF, was marketed in Germany under the brand name “Fumaderm” for the treatment of moderate-to-severe plaque psoriasis (Burness and Deeks, 2014; Tintore and Sastre-Garriga, 2016). Over the years, researchers discovered that DMF exerts immunomodulatory and anti-inflammatory effects, particularly through the modulation of the nuclear factor erythroid 2-related factor 2 (Nrf2) (To et al., 2015) and nuclear factor-kappa B (NF-κB) signaling pathway (Peng et al., 2012). By activating nuclear factor erythroid 2-related factor 2 (Nrf2), DMF enhances the cellular antioxidant response, reducing oxidative stress, while its inhibition of NF-κB leads to suppression of pro-inflammatory cytokine production (Deeks, 2016; Tintore and Sastre-Garriga, 2016). These mechanisms of action led to interest in DMF for treating neuroinflammatory diseases such as multiple sclerosis (MS) (Deeks, 2016; Tintore and Sastre-Garriga, 2016). Clinical trials demonstrated that DMF significantly reduced relapse rates and the formation of new brain lesions in patients with relapsing-remitting MS (RRMS), and therefore, the U.S. Food and Drug Administration (FDA) approved DMF (marketed as Tecfidera) in 2013 for the treatment of RRMS (Serra and Fox, 2013). DMF became the first oral fumarate-based therapy for MS, offering patients a convenient alternative to injectable treatments. Since then, DMF has also drawn significant attention for its potential anticancer properties (Al-Jaderi and Maghazachi, 2016), prompting ongoing research into its repurposing as a therapeutic agent beyond autoimmune and neuroinflammatory disorders.

Recently, especially in the past decade, a growing number of studies have been conducted using DMF as an anticancer agent, including its single use and in combination with other chemotherapies. Since DMF is already an approved drug, we believe it holds great promise once approved for its new indications, specifically for cancer, either as a single agent or in combination. Therefore, in this review, we attempted to have a brief overview of the basic, preclinical or clinical studies (if available) using DMF for cancer treatment. While we primarily focused on studies published in the past decade, from 2015 to 2024, to achieve broader coverage, we will also include those published in 2025 (as of the end of July).

## 2 DMF as a single agent for cancer treatment

DMF is known for its anti-inflammatory activity via the inhibition of NF-κB; thus, numerous studies have attempted to identify cancer types that heavily rely on NF-κB, and breast cancer is one of these cancer types. Kastrati et al. (2016) found that the NF-κB pathway promotes survival, migration, invasion, angiogenesis, stem cell-like properties, and resistance to therapy of aggressive breast cancers, and they hypothesized that DMF could, therefore, hold promise in suppressing breast cancer via inhibiting NF-κB (Kastrati et al., 2016). Indeed, they found that DMF (IC_50_ value approximately 20 μM) effectively blocks NF-κB activity in multiple breast cancer cell lines, including MCF-7, T47D and the ER^+^/Her2^+^ cell line BT474, and it abrogates NF-κB-dependent sphere formation, a key assay assessing the stem-like property. Mechanistically, DMF (20 μM, 2–3 h) prevents p65 nuclear translocation and attenuates its DNA binding activity, as validated by a designed clickable DMF-based probe. Finally, DMF (30 mg/kg, daily) inhibits cell proliferation and significantly impairs the growth of xenograft tumors by triple-negative breast cancer (TNBC) MDA-MB-231 cells, with an inhibition rate of ∼50%. In this work, they also confirmed that the double bond in fumarate is essential for targeting NF-κB, since dimethyl succinate (Figure 1B), the inactive analog of DMF that lacks the electrophilic double bond of fumarate, is unable to inhibit NF-κB activity (Kastrati et al., 2016). This result may provide critical information for developing DMF derivatives and/or analogs that possess higher potency than DMF, warranting further study. Despite recent progress, curative therapies for cutaneous T-cell lymphoma (CTCL) remain elusive, underscoring the need for novel treatment strategies with enhanced efficacy and fewer side effects. A hallmark of CTCL is its resistance to cell death, which is attributed to constitutive NF-κB activation, making this pathway an attractive and selective therapeutic target. DMF (30 µM), as an NF-κB inhibitor, selectively induced cell death in patient-derived CTCL cells and cell lines while sparing healthy donor T cells, with its cytotoxicity linked to NF-κB inhibition. In two CTCL xenograft mouse models with different tumor localizations, DMF (20 or 30 mg/kg) treatment significantly delayed tumor growth, prevented distant metastases, and enhanced apoptosis in both primary tumors and metastatic lesions (Nicolay et al., 2016). In malignant melanoma B16BL6 cells with NF-κB activation, DMF was effective in inhibiting proliferation (100 μM), metastasis (50 and 100 μM) *in vitro*, and reducing tumor growth in the B16BL6 cells xenograft model *in vivo* (10 and 30 mg/kg, daily). The mechanism study suggested that DMF *in vivo* prevented the nuclear translocation of NF-κB, without affecting the phosphorylation levels of the inhibitor of κB (IκB). At the protein level by examining the tumor tissues, DMF reduced the expression of matrix metalloproteinases (MMPs) and very late antigens (VLAs). DMF appears to induce the canonical cell death mediated by the inhibitor of apoptosis protein (IAP) Survivin and B-cell lymphoma-extra large (Bcl-xL), suggesting DMF may be a potential therapeutic agent for metastatic melanoma (Takeda et al., 2020). It is also reasonable to predict that DMF can suppress any signal pathways or proteins that have interactions with NF-κB activation.

A study showed that NF-κB stimulated the expression of antiapoptotic proteins such as inhibitors of apoptosis (IAPs) and FLICE-like inhibitory proteins (cFLIPs) (Schroeder et al., 2017), both of which were universally upregulated in cancers but not in normal cells (Peery et al., 2022; Cui et al., 2023). Meanwhile, thioredoxin-1 (Trx1), a major regulator of NF-κB transcriptional activity, as well as members of IAPs and cFLIP, can be inhibited or induced degradation upon DMF (50 μM) treatment in T-cell lymphomas and leukemias, via the Michael addition of the double bond in DMF (Schroeder et al., 2017). A clinical study showed that in patients with cutaneous T-cell lymphoma, DMF treatment can activate caspase-3 and caspase-9, two downstream proapoptotic proteins of IAPs, in T cells, thereby inducing cell death predominantly in malignant or activated T cells (Schroeder et al., 2017). In BC3 and BCBL-1 PEL (a rare B cell lymphoma) cell lines, DMF (20 and 50 µM for 24 h) was also found to suppress p-STAT3, in addition to activating Nrf2, reducing ROS and the expression of proinflammatory cytokines IL-6 and IL-10 which are essential for PEL cells (Gonnella et al., 2022). Specifically, DMF appeared to be more sensitive in the ERK1/2- and autophagy-activated fresh BC3 PEL cells than in fresh PEL cell lines (Gonnella et al., 2022), suggesting it may have similar effects in other cancer cell lines with the same patterns as the fresh PEL cells.

In addition to targeting NF-κB, DMF appears to be active in targeting other members in regulating inflammation, such as Regnase-1, which is a ribonuclease and a tumor suppressor gene mutated in colorectal cancer tissue and is related to poor prognosis (Okabe et al., 2023). Iguchi et al. (2025) validated Regnase-1’s effects in a geoengineering mouse model, with significantly reduced tumor growth when this gene was absent, accompanied by elevated levels of phosphorylated extracellular signal-regulated kinase (ERK) (Iguchi et al., 2025). Further study indicated that Nfkbiz, a mediator of IL-17, is Regnase-1’s target, and the absence of Regnase-1 may lead to the activation of IL-17, which then triggers pro-cancer inflammation. DMF (25 µM) has been shown to activate Regnase-1, and oral treatment of dimethyl fumarate (dissolved in drinking water at a concentration of 0.05%), suppressed tumor growth of HT29 cells xenograft model, downregulated Nfkbiz, and pERK activation, without causing any systemic adverse effects, including body weight loss and confirming DMF’s effects on Regnase-1 and suppressing colon tumor (Iguchi et al., 2025).

More details have been revealed that DMF (higher than 500 µM) could suppress the proliferation and induce apoptosis via enhancing proapoptotic Bax, caspase 3, and BID, while decreasing antiapoptotic Bcl-2 in oral squamous cell carcinoma (OSCC) CAL27, HSC-2, and HSC-3 cells, as shown in Basilotta et al.’s work published in 2023 (Basilotta et al., 2023). DMF (0.5, 1, and 10 mM) was also confirmed to be a critical player in regulating oxidative stress by modulating the antioxidative enzymes, including MnSOD and HO-1, which subsequently leads to the overproduction of ROS, ultimately causing cell death in CAL27 cells. Importantly, in this work, DMF (1 mM and 10 mM, 48 h) inhibited the migratory ability of tumor cells by modulating N-cadherin and E-cadherin, two markers of epithelial-mesenchymal transition (EMT). These mechanisms worked together to lead to the inhibited tumor growth of xenograft model by DMF at a daily dose of 100 mg/kg (Basilotta et al., 2023). It should be noted that DMF is relatively less potent (25-fold) in OSCC cells than in the other cancer cells discussed above, suggesting that DMF’s sensitivity is cell type-dependent and requires further validation. Non-small cell lung cancer (NSCLC) remains a leading cause of cancer-related mortality, underscoring the need for novel therapeutic strategies. In a recent preclinical study, the efficacy of DMF was evaluated in NSCLC models. Using *in vitro* cell viability assays and *in vivo* graft models with both immunocompetent and immunodeficient mice, DMF (30 mg/kg, administered orally, 5 times a week) was shown to inhibit tumor progression and growth without causing significant apparent toxicity in three animal models including A549, KLN205 and LLC1 xenograft models (Rupp et al., 2022). *In vitro*, DMF (1–100 μM) reduced cancer cell proliferation, indicating a direct antitumor effect (Rupp et al., 2022).

Another study showed that DMF (50 and 100 μM for 24 and 48 h) induced apoptosis of adult T-cell leukemia (ATL) MT-1 and MT-2 cells mediated by cleaved poly ADP-ribose polymerase (PARP) (Maeta et al., 2022). Interestingly, DMF (50 and 100 μM) suppressed the constitutive activation of both canonical and non-canonical NF-κB pathways in MT-2 cells, while it only inhibited the non-canonical NF-κB pathway in MT-1 cells. In addition to activating PARP which suggested that DMF may cause DNA damage, DMF also suppressed antiapoptotic cIAP, and the phosphorylation of STAT3 (Maeta et al., 2022). It is noted that DMF acted differently in these 2 cells, and was more sensitive in MT-2 cells. Further molecular signaling studies are needed to guide the use of DMF across different cell types.

DMF is found to form a covalent bond with the oncoprotein ZNF217, as confirmed by proteomics in Sharma et al.’s work (Sharma et al., 2023). In ER^+^ MCF-7 breast cancer cells, ZNF217 appears to play important role in promoting stem-like properties, survival, proliferation, and invasion. Since DMF can directly bind to ZNF217, it (50 µM and 100 μM, for 2 or 4 h) suppressed ZNF217s targeted genes *ERBB3* and *SNAI2*, accompanied by the inhibition of ZNF217-mediated phenotypes, which could be reversed, at least partially, by ZNF217 knockdown, supporting DMF’s targeting effect (Sharma et al., 2023). In a xenograft model of MCF-7 HER2 cells overexpressing ZNF217, DMF (30 mg/mouse) significantly inhibited tumor growth (Sharma et al., 2023).

Kirsten rat sarcoma viral oncogene homolog (KRAS) mutations, particularly G12 V, are among the most frequent alterations in adult carcinomas and are known to activate the Nrf2-driven antioxidant response (Tao et al., 2014). DMF (100 μM, 72 h) exerts preferential cytotoxicity against KRAS-mutated cancer cells by inhibiting the Nrf2/DJ-1 pathway (Bennett et al., 2018). Using *in vitro* assays, DMF (100 μM, 24 h) significantly increased cell death, ROS generation, and glutathione (GSH) depletion in KRAS*G12 V-mutated cell lines, including patient-derived models and Caco-2 cells overexpressing KRAS*G12V, compared to wild-type controls (Bennett et al., 2018). In contrast, DMF showed minimal cytotoxicity in non-tumorigenic cells, including ARPE-19, 3T3 fibroblasts, and mouse bone marrow cells, where it instead activated Nrf2, reduced ROS, and elevated GSH levels. Notably, DJ-1 downregulation impaired Nrf2 function in malignant but not in non-malignant cells, suggesting a tumor-specific vulnerability. These findings indicate that KRAS-mutant cancer cells are selectively sensitive to Nrf2 inhibition by DMF, supporting its potential as a targeted therapeutic agent in KRAS-driven malignancies (Bennett et al., 2018).

Another interesting study showed that DMF (20–200 μM), but not its metabolite mono-methyl fumarate (MMF, as shown in Figure 1A), induced necroptosis (but not apoptosis) in colon cancer HCT116 and CT26 cells, as evidenced by necrostatin-1 sensitivity, LDH and HMGB1 release, and ultrastructural changes. Interestingly, DMF only significantly reduces the cell survival at 50 μM or higher, consistent with the previous studies. DMF (100 μM) treatment led to GSH depletion, increased ROS, and activation of JNK, p38, and ERK MAPK pathways, all of which were attenuated by antioxidants GSH and NAC. While DMF also promoted autophagy in several GI cancer cell lines, autophagy inhibition did not rescue cell viability, suggesting it is not the primary mechanism of cytotoxicity (Xie et al., 2015).

In addition to affecting certain signal pathways or target proteins, DMF also was able to impact energy metabolism, such as oxidative phosphorylation (OXPHOS), or aerobic glycolysis, as shown in Chen et al.’ work published in 2021 (Chen et al., 2021). In pancreatic cancer ANC-1, CFPAC-1, Patu-8988, and Miapaca-2 cells, DMF (25–200 µM) suppressed both mitochondrial respiration, determined by the oxygen consumption rate (OCR), and aerobic glycolysis, determined by extracellular acidification rate (ECAR), which could be reversed by treatment with L-cysteine and N-acetyl-L-cysteine (NAC), two ROS scavengers and antioxidants (Chen et al., 2021). Their virtual docking study suggested that MTHFD1 (Methylenetetrahydrofolate dehydrogenase 1) might be DMF’s target, which negatively correlated with the prognosis of pancreatic patients (Chen et al., 2021), warranting further experimental validation.

DMF is also able to induce cell cycle arrest at G1 in primary human dermal lymphendothelial cells (DLEC) via downregulating Cyclin D1 and Cyclin A expression and upregulating p21 (Valesky et al., 2016). Furthermore, DMF is also highly efficient in suppressing the proliferation of Merkel cell carcinoma (MCC) MCC13, MCC14.2, and MCC26 cells (Gambichler et al., 2023), neuroblastoma NB-EBC1 cells overexpressing MYCN, a member of the Myc oncogene family (Wang et al., 2018), cervical cancer HeLa cells (via activating caspase-3 and PARP) (Han and Zhou, 2016), CT26, HT29, and HCT116 colon cancer cells (Ma et al., 2016), suggesting it is a broad-spectrum anticancer agent.

## 3 DMF as a chemosensitizer

Recent studies have highlighted the potential of DMF as a promising adjunct in cancer therapy, as we discussed above. Beyond its well-known anti-inflammatory and antioxidant properties, DMF has shown the ability to sensitize cancer cells to various chemotherapeutic agents, enhancing their anticancer efficacy. Mechanistically, DMF can modulate redox balance, disrupt pro-survival signaling pathways, and promote apoptosis in tumor cells.

TNBC is the most aggressive subtype of breast cancer, characterized by poor prognosis and limited treatment options. The constitutive activation of NF-κB signaling in TNBC has been linked to tumor growth, survival, and therapeutic resistance, indicating that NF-κB may be a potential therapeutic target. Tsurushima et al.’s study showd that DMF (1–50 μM) induced apoptosis in TNBC cells, including MDA-MB-231 and BT-549 lines, at concentrations that were non-toxic to normal mammary epithelial cells (Tsurushima et al., 2022). DMF treatment inhibited NF-κB nuclear translocation and downregulated key anti-apoptotic proteins, such as Survivin, XIAP, Bcl-xL, and Bcl-2. Moreover, DMF (1, 5, and 10 μM) enhanced the apoptosis-inducing effects of conventional chemotherapeutic agents such as paclitaxel and doxorubicin, suggesting its potential as a chemosensitizer (Tsurushima et al., 2022). Further animal models are needed to validate DMF’s sensitizing effects to these two drugs.

In another study using hormone-nonresponsive AMJ13 breast cancer cell line, DMF demonstrated synergistic effects when combined with photodynamic therapy (PDT), a minimally invasive approach that induces tumor cell death via the overproduction of ROS. In this study, DMF (2.5 and 1.25 μg/mL) was combined with PDT using aminolevulinic acid (ALA) and He-Ne laser irradiation against the hormone-nonresponsive AMJ13 breast cancer cell line (Al-Khafaji et al., 2024). The co-treatment significantly enhanced cytotoxicity compared to either treatment alone, and combination index analysis confirmed a synergistic interaction between DMF and PDT (Al-Khafaji et al., 2024).

DMF in normal cells is known to stimulate Nrf2, thereby suppressing oxidative stress. In cancer cells, the effects of this protein, whether activation or inactivation, depend on its concentration. As shown in Saidu et al.’s work (2017), DMF at 25 μM activates the Nrf2 antioxidant pathway; however, at higher than 25 μM, it could inhibit the nuclear translocation of Nrf2, and subsequently, suppress Nrf2’s downstream proteins that resist oxidative stress in OVCAR3 cells (Saidu et al., 2017). A rescue experiment further validated this result by overexpressing Nrf2, and DJ-1, a Nrf2 protein stabilizer, in OVCAR3 cells, leading to apoptosis. DMF (20 mg/kg, daily) could suppress the tumor growth of the CT26 cells xenograft model alone or combined with paclitaxel (20 mg/kg, three times/week). The combination showed higher tumor-inhibitory effects than DMF or paclitaxel alone, suggesting DMF’s sensitization to paclitaxel (Saidu et al., 2017). Furthermore, DMF’s on-target effects on Nrf2 and DJ-1 were also confirmed in this animal model.

Hepatocellular carcinoma (HCC) remains a major global health challenge due to its poor prognosis, limited long-term response to current therapies, and high incidence of resistance. A recent study showed that DMF (50–200 μM) is a promising agent that suppresses HCC growth by targeting the Nrf2-Bcl-xL signaling axis, a pathway associated with poor outcomes in certain HCC patients (Faleti et al., 2025). DMF (150 μM for 24 or 48 h) downregulates this pathway, inducing mitochondrial stress and apoptosis both *in vitro* (Huh7 and HepG2 cells) and *in vivo* (Huh7 cells xenograft model). Notably, overexpression of Nrf2 or Bcl-xL reversed DMF’s antitumor effects, confirming the pathway’s central role. Furthermore, DMF (30 mg/kg, daily) enhanced the efficacy of sorafenib (30 mg/kg, daily), a standard HCC treatment, without adding toxic effects to reduce the body weight or impact the renal and hepatic functions of treated mice, resulting in an almost complete halt of tumor growth (Faleti et al., 2025). Acute myeloid leukemia (AML) is a highly aggressive hematological malignancy lacking effective therapies for most patients. Vitamin D derivatives (VDDs), such as 1,25-dihydroxyvitamin D_3_ (1,25D_3_) and its analogs, are known to induce differentiation in AML cells; however, their clinical application is limited by calcemic toxicity at therapeutic doses. DMF (50 μM) can synergistically enhance the pro-differentiation effects of VDDs in AML cell models (Nachliely et al., 2019). DMF significantly amplified the activity of both 1,25D_3_ and the potent analog PRI-5202 by upregulating vitamin D receptor (VDR) and Nrf2 signaling, resulting in increased expression of their downstream target genes (Nachliely et al., 2019). Importantly, in an AML xenograft model, the combination of DMF (0.6 mg/mouse) and PRI-5202 (0.25 μg/mouse), a derivative of 1,25D_3_, led to a strong cooperative inhibition of tumor growth without causing toxicity (Nachliely et al., 2019).

Clear cell renal cell carcinoma (ccRCC) is the most aggressive form of kidney cancer with limited effective treatment options. Recent findings reveal that DMF (0–100 μM) inhibits ccRCC cell proliferation by targeting hepatocyte nuclear factor 1β (HNF1B). DMF (50 μM, 12 h) covalently modifies HNF1B at cysteine residues, promoting its proteasomal degradation, which can be reversed by a proteasome inhibitor MG132 (10 μM, 12 h). Since HNF1B stabilizes Yes-associated protein (YAP), its loss induced by DMF leads to decreased YAP levels and downregulation of proliferative target genes. Moreover, oral DMF (30 mg/kg, daily) enhances the antitumor effect of sunitinib (20 mg/kg, every other day) *in vivo* in the 786-O cells xenograft model, supporting its potential as a novel therapeutic strategy for ccRCC through disruption of the HNF1B–YAP axis (Dai et al., 2025).

DMF also regulates YAP in melanoma, which is a highly aggressive skin cancer with limited responsiveness to conventional therapies. While BRAF inhibitors (BRAFi) such as vemurafenib can offer temporary clinical benefits in patients with BRAF-mutant melanoma, the development of resistance remains a major therapeutic hurdle. Combining DMF (25–150 µM *in vitro*, and 6 mg/kg *in vivo*) with vemurafenib (2 µM *in vitro* and 25 mg/kg *in vivo*) enhances antitumor efficacy compared to either agent alone. This combination (2 µM vemurafenib and 50 µM DMF) induces robust cell death by suppressing the transcriptional activity of Nrf2, leading to elevated ROS, and by downregulating YAP. In addition, the combination attenuates AKT/mTOR/ERK signaling by reducing phosphorylation of AKT, 4EBP1, P70S6K, and ERK. Transcriptomic analysis further revealed that this dual treatment downregulates thousands of genes across multiple oncogenic pathways (Li et al., 2022).

Dietary fatty acids have emerged as important modulators of immune function, and in a recent study, metabolomic profiling of cancer patients receiving Vγ9Vδ2-T cell therapy revealed that circulating levels of palmitic acid (PA) and oleic acid (OA) correlate with therapeutic efficacy (Zhang et al., 2025). Mechanistically, PA impairs γδ T cell antitumor activity by inducing excessive IFNγ secretion, which triggers pyroptotic cell death, whereas OA restores function by reducing IFNγ levels and preventing pyroptosis. Importantly, pharmacological interventions targeting IFNγ by DMF (50 mg/kg) effectively blocked pyroptosis and restored γδ T cell cytotoxicity (Zhang et al., 2025).

Oncolytic virotherapy, particularly using genetically modified herpes simplex virus-1 (oHSV-1), has shown considerable promise as a cancer immunotherapy, with the FDA approval of oHSV-1 expressing GM-CSF for melanoma marking a significant milestone. However, limited responsiveness in certain tumor types has posed a challenge. Recent evidence suggests that DMF (100 and 150 µM) has the potential to enhance the efficacy of oHSV-1. Preclinical studies reveal that DMF (200 mg/kg, oral gavage, three times every other day) significantly boosts viral replication and oncolysis in the CT26.wt and 4T1colon carcinoma cells xenograft models, while sparing normal tissues (Selman et al., 2018). Mechanistically, this enhancement is mediated by suppression of the type I IFN response, a key barrier to effective oncolytic virus spread. Notably, combination treatment with DMF and oHSV-1 improves therapeutic outcomes in aggressive murine cancer models (Alwithenani et al., 2023), suggesting the potential for repurposing DMF as a chemosensitizer for oncolytic virotherapy, which warrants further clinical evaluation in cancer patients. Similarly, DMF (150 and 250 µM) also enhances the efficacy of oncolytic virotherapy by promoting viral infection and spread in resistant tumors through suppression of type I IFN signaling, primarily via inhibition of NF-κB nuclear translocation (Selman et al., 2018).

DMF, through its active metabolite MMF (Figure 1A), demonstrates promising anticancer potential when used in combination with other agents. In particular, MMF (5 μM) synergizes with ruxolitinib (1 μM) to induce apoptosis in a wide range of cancer cell types, including those of the brain, breast, lung, and ovarian cancers, such as H1975 NSCLC cells, BT474 and GBM12 cells, as well as SUM149 and BT474 cells (Tavallai et al., 2016). This combination disrupts multiple key survival pathways such as ERK1/2, AKT, and STAT3/5, downregulates anti-apoptotic proteins (MCL-1, Bcl-xL, SOD2, Trx), and enhances pro-apoptotic signaling (BIM expression and BAD dephosphorylation). Notably, the drug combination significantly increases oxidative stress, and antioxidant overexpression can block its lethal effects, indicating a redox-based mechanism (Tavallai et al., 2016). Furthermore, DMF has shown the capacity to sensitize cancer cells to paclitaxel (10 nM) or docetaxel (10 nM) (Tavallai et al., 2016), warranting further *in vivo* study.

DMF and its active metabolite MMF have shown potential in enhancing natural killer (NK) cell-based immunotherapy. DMF at 100 μM upregulated chemokines receptor 10 (CCR10) expression on IL-2-activated human NK cells, promoting their migration toward tumors that secrete CCR10 ligands such as CCL27 and CCL28 (Maghazachi et al., 2016). Importantly, the enhanced chemotaxis is coupled with increased NK cell cytotoxicity against tumor targets, an effect that is CCR10-dependent. This suggests that DMF can be used to functionally prime NK cells *in vitro* for improved trafficking and killing of specific cancers, including melanoma, squamous cell carcinoma, and colorectal cancer (Maghazachi et al., 2016).

DMF’s effects on EMT can be used to overcome resistance mediated by the induction of EMT. E.g., oxaliplatin and 5-fluorouracil (5-FU) induce EMT in KRAS G13D-mutated colon cancer LoVo and DLD-1 cells, contributing to enhanced migration and drug resistance (Hoshida et al., 2025). This process is driven by activation of the KRAS/ERK/NF-κB signaling pathway. Notably, DMF (5 μM) effectively suppressed EMT induction by oxaliplatin and 5-FU, thereby enhancing the sensitivity to oxaliplatin and 5-FU in L-OHP- and 5-FU-induced EMT cells (Hoshida et al., 2025).

## 4 DMF as a protector against chemotherapy-induced toxicities

The clinical benefits of chemotherapies are often limited by significant adverse effects that impact patient quality of life and treatment adherence. There is growing interest in identifying supportive agents that can alleviate these toxicities without reducing therapeutic efficacy. DMF has emerged as a promising candidate due to its anti-inflammatory and cytoprotective properties. Recent studies suggest that DMF may help reduce chemotherapy-induced side or adverse effects while preserving the effectiveness of anticancer therapies. Therefore, in this section, we summarize recent studies that utilize DMF as an ameliorator to counteract the toxic effects of chemotherapies.

DMF has shown neuroprotective properties, which was able to alleviate oxaliplatin-induced peripheral neuropathy without compromising its anticancer efficacy, as shown in Miyagi et al.’s study (Miyagi et al., 2019). DMF (0.3–10 µM *in vitro*, 100 and 200 mg/kg *in vivo*) significantly reduced oxaliplatin-induced mechanical allodynia and axonal degeneration in the sciatic nerve, though it had a limited effect on cold hyperalgesia. Importantly, DMF did not worsen systemic side effects such as body weight loss or bone marrow suppression, nor did it interfere with oxaliplatin’s anti-tumor activity in multiple cancer cell lines and tumor-bearing mice. MMF (0.3–10 µM *in vitro*) also showed similar effects as DMF (Miyagi et al., 2019).

Doxorubicin (DOX), although widely used as a chemotherapeutic agent, is clinically limited by its cardiotoxicity, which is primarily mediated by oxidative stress and apoptosis. DMF (10 and 20 μM) could protect against DOX-induced cardiac injury using both neonatal rat cardiomyocytes (NRCMs) *in vitro* and a DOX-induced cardiotoxicity model *in vivo* at 40 and 80 mg/kg (Hu et al., 2022). DMF significantly improved cell viability and morphology in NRCMs and alleviated DOX-induced cardiac damage in rats, as evidenced by reduced CK-MB and LDH levels, improved survival and cardiac function, and ameliorated histopathological changes. Mechanistically, DMF suppressed oxidative stress by decreasing MDA and increasing GSH, SOD, and GSH-px levels, while also inhibiting apoptosis through modulation of Bax, Bcl-2, and cleaved caspase-3. These protective effects were dependent on Nrf2 activation, as DMF promoted Nrf2 nuclear translocation and upregulated its downstream antioxidant gene Hmox1, whereas Nrf2 silencing abrogated these benefits. Notably, DMF did not interfere with the cytotoxicity of DOX in tumor cells, suggesting its cardioprotective effect does not compromise anticancer efficacy (Hu et al., 2022).

Cyclophosphamide (CP)-induced acute cystitis, a common yet painful complication in cancer patients, is largely driven by oxidative stress and inflammation in bladder tissue. DMF (100 or 300 mg/kg/day) demonstrated significant uroprotective effects, particularly at higher doses, by preserving bladder contractility, reducing vascular permeability, and restoring GSH levels (Barut et al., 2025). Though DMF did not significantly lower TNF-α levels, its antioxidant properties contributed to mitigating bladder damage. Notably, when combined with CP, DMF (1, 10, and 100 µM) also enhanced the cytotoxic effects in SH-SY5Y cells, indicating a potential synergistic role of both in eliminating toxic effects while enhancing cytotoxic effects toward cancer cells (Barut et al., 2025).

Docetaxel is widely employed in cancer treatment but is frequently associated with dose-limiting toxicities, particularly myelotoxicity and peripheral neuropathy. In a Wistar rat model established to simultaneously mimic both adverse effects, DMF (100 mg/kg/week) demonstrated significant neuroprotective effects, alleviating docetaxel-induced hyperalgesia and preserving nerve fiber density in the sciatic nerve (Cubides-Cely et al., 2025). While its effect on neutropenia was limited under standard dosing, a modified regimen with pre-treatment showed a trend toward hematologic protection and even reduced vibrissae loss. Importantly, combination studies in prostate cancer cell lines confirmed that DMF does not compromise docetaxel’s anticancer efficacy. In fact, synergistic interactions were observed in many dosing ratios, e.g., docetaxel(5): DMF (1) (Cubides-Cely et al., 2025).

## 5 Discussion

**FIGURE 2:**
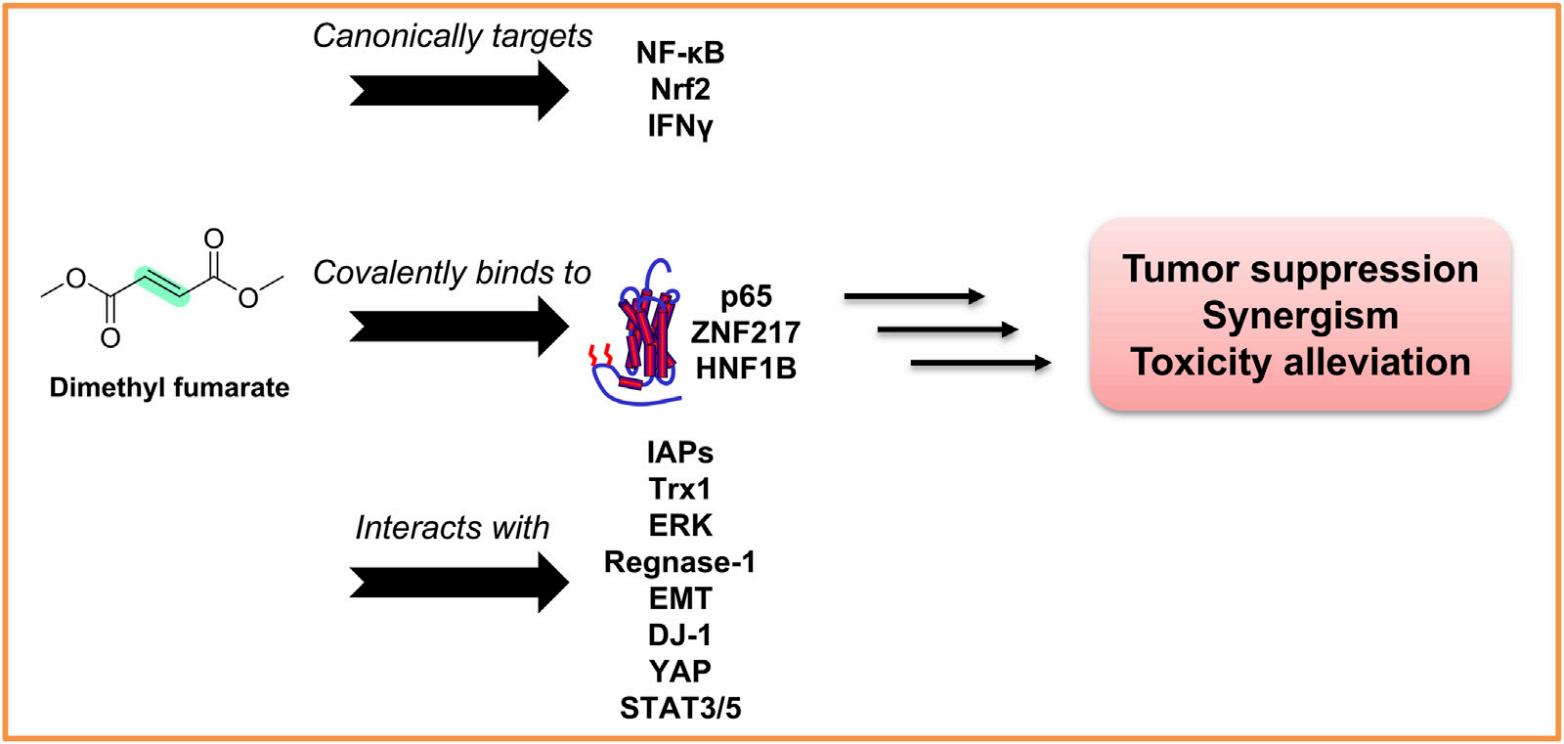
DMF as an anticancer agent via polypharmacology. DMF canonically targets signaling pathways including NF-κB, Nrf2, and IFNγ, while covalently binding to redox- and transcription-related proteins such as p65, ZNF217, and HNF1B through Michael addition. It also interacts with a wide spectrum of cellular regulators including IAPs, Trx1, ERK, Regnase-1, EMT factors, DJ-1, YAP, and STAT3/5. These multimodal interactions contribute to tumor suppression, synergistic enhancement with chemotherapeutic agents, and attenuation of drug-induced toxicities.

The above information suggests that DMF is a potential and powerful anticancer agent in single or in combination for some drug-resistant cancers via multiple mechanisms, as summarized in Figure 2.

According to the updated information on clinicaltrials.gov (https://clinicaltrials.gov/), six clinical trials have been completed, with no further scheduled trials. More studies are needed to test DMF alone or in combination in patients who are resistant to certain treatments.

DMF is an electrophilic compound capable of forming covalent bonds with nucleophilic residues, predominantly through thiol (–SH) groups found in cysteine residues of proteins (Satoh and Lipton, 2017). This mechanism underlies its ability to modify key redox-sensitive proteins involved in cellular stress responses. However, due to the widespread presence of thiol groups across the proteome, it is reasonable to predict that DMF’s reactivity may not be highly selective for a single target. As a result, DMF could potentially interact with a broad range of cellular proteins as we reviewed above. While this non-selective reactivity contributes to its broad pharmacological actions, including anti-inflammatory and anti-cancer effects, it also raises concerns regarding off-target effects and potential cytotoxicity in certain conditions. Therefore, understanding the thiol-reactivity profile of DMF and identifying its key functional protein targets remain critical for optimizing its therapeutic use and minimizing unintended adverse effects.

DMF appears to have dual functions with respect to Nrf2, activating or deactivating it in different cancer cells (Izumi and Koyama, 2024). At low concentrations (<25 μM), DMF activates Nrf2 by modifying KEAP1, promoting antioxidant gene expression and cytoprotection, as seen in breast cancer models where it reduces tumor invasion via macrophage modulation. At higher concentrations (>25 μM), DMF inhibits Nrf2, particularly in cancers with high Nrf2 activity (e.g., KRAS-mutated lung or ovarian cancers), by downregulating DJ-1, leading to increased oxidative stress and cytotoxicity. This dose- and context-dependent duality highlights DMF’s potential for both cancer prevention (via Nrf2 activation) and treatment (via Nrf2 inhibition in NRF2-dependent tumors), necessitating the development of tailored therapeutic strategies based on cancer type and genetic profile.

In most cancer studies, DMF or its active metabolite MMF is used at *in vitro* concentrations ranging from 25 to over 100 µM to elicit anticancer effects. However, pharmacokinetic data in humans indicate that the peak plasma concentration (C_max_) of MMF after standard oral dosing (e.g., 240 mg twice daily for multiple sclerosis) is approximately 6–15 µM (the number may also vary), with a short half-life of less than 1 hour and no accumulation upon repeated administration (Booth et al., 2014). This suggests that the concentrations commonly used *in vitro* may not be achievable or sustainable in patients without posing safety concerns. While some animal studies have used higher oral doses to demonstrate efficacy, toxicity has been reported at elevated doses, indicating a narrow therapeutic window (Ahmadi-Beni et al., 2019). Therefore, this discrepancy raises concerns about clinical translatability, emphasizing the need for further studies to confirm efficacy at physiologically relevant concentrations, explore alternative delivery strategies, or evaluate synergistic combinations that allow for lower, safer dosing.

## 6 Conclusion

DMF is an approved drug for multiple sclerosis, which has also demonstrated promising anticancer effects alone or in combination with other conventional, targeted or immunotherapies. Importantly, as a cytoprotective agent, DMF is also able to alleviate toxic effects caused by chemotherapy. Further clinical studies are needed to advance its application.
